# Draft Genome Sequences of 18 Oral Streptococcus Strains That Encode Amylase-Binding Proteins

**DOI:** 10.1128/genomeA.00510-15

**Published:** 2015-05-21

**Authors:** Amarpreet Sabharwal, Yu-Chieh Liao, Hsin-Hung Lin, Elaine M. Haase, Frank A. Scannapieco

**Affiliations:** a Department of Oral Biology, University at Buffalo, State University of New York, Buffalo, New York, USA; b Division of Biostatistics and Bioinformatics, Institute of Population Health Sciences, National Health Research Institutes, Zhunan, Miaoli, Taiwan

## Abstract

A number of commensal oral streptococcal species produce a heterogeneous group of proteins that mediate binding of salivary α-amylase. This interaction likely influences streptococcal colonization of the oral cavity. Here, we present draft genome sequences of several strains of oral streptococcal species that bind human salivary amylase.

## GENOME ANNOUNCEMENT

Streptococcus species are primary colonizers of the tooth surface facilitating the formation of dental plaque. Some oral streptococcal species bind α-amylase, the most abundant protein in human saliva. The ability to bind salivary amylase may provide a selective advantage within the oral microbial niche. Investigations into the mechanism of amylase binding using an amylase ligand-binding assay (1, 2) revealed a variety of amylase-binding proteins (ABPs) (3–6). During the course of our recent studies of ABPs, we wished to rapidly determine the genetic sequence encoding the ABPs identified by N-terminal sequencing. To facilitate these studies, the genomes of 18 strains were sequenced, assembled, and annotated, ultimately to study the evolution of ABPs and other proteins in oral streptococci.

Each streptococcal strain studied originated from a site in the human oral cavity. Genomic DNA was extracted from overnight cultures, as previously described (7), treated with RNase A/T1 mix (Thermo Fisher Scientific, Inc.) and further purified using the QIAamp DNA minikit (Qiagen) for high-throughput sequencing. DNA libraries were prepared using the TruSeq DNA multiplexed library preparation kit v2.0 (Illumina). DNA sequencing was performed in rapid 150-cycle paired-end mode in a single lane using an Illumina HiSeq 2500 analyzer (http://www.buffalo.edu/bioinformatics.html), which achieved 150-bp read lengths and over 100× coverage. The paired-end sequencing reads were checked for quality, *de novo* assembled, and annotated using MyPro, a software pipeline for prokaryotic genomes (8). This software is available for download at http://sourceforge.net/projects/sb2nhri/files/MyPro/. With available reference genomes, eight of the 18 assemblies were post-assembled (align, order, and connect) using MyPro to generate superior draft genomes as shown in Table 1. The mean numbers of contigs obtained for the 8 and the remaining 10 strains (without post-assembly) were 6.9 and 13.6, respectively. Similarly, the mean *N*_50_ values were 1 Mb and 620 Kb, respectively. After manually excluding contaminant and phage sequences, the annotated sequences were submitted to NCBI. Detailed commands conducted for the 18 draft assemblies can be found on the website of MyPro.

**TABLE 1:** Characteristics of 18 oral streptococcus draft genomes

Strain name	No. of contigs	Size (Mb)	G+C content (%)	No. of CDSs	No. of rRNAs	No. of tRNAs	Accession no.
*S. cristatus* CC5A^a^	7	2.03	42.7	1,924	13	66	JYGJ00000000
*S. cristatus* CR3^a^	5	2.00	42.6	1,890	15	60	JYGK00000000
*S. gordonii* G9B^a^	2	2.20	40.5	2,085	8	57	JYGL00000000
*S. mitis* COL85/1862	11	1.90	41.2	1,840	6	54	JYGM00000000
*S. mitis* NCTC10712	7	2.19	40.6	2,050	12	66	JYGN00000000
*S. mitis* OP51	10	1.84	41.4	1,774	21	58	JYGO00000000
*S. mitis* OT25^a^	3	1.92	40.1	1,826	12	63	JYGP00000000
*S. mitis* SK137^a^	7	1.98	40.2	1,876	12	62	JYGQ00000000
*S. mitis* SK141	5	1.86	41.1	1,810	4	51	JYGR00000000
*S. mitis* SK145^a^	8	1.97	40.0	1,860	9	62	JYGS00000000
*S. mitis* UC921A	11	1.79	39.2	1,758	13	69	JYGT00000000
*S. mitis* UC5873	10	1.84	41.2	1,796	5	51	JYGU00000000
*S. mitis* UC6950A	29	2.02	39.8	1,900	9	54	JYOV00000000
*S. parasanguinis* MGH413^a^	2	2.10	42.0	1,957	5	58	JYOW00000000
*S. salivarius* KB005^a^	21	2.29	39.6	2,085	13	54	JYOX00000000
*S. salivarius* UC3162	23	2.16	40.1	1,938	26	70	JYOY00000000
*S. sanguinis* I141	10	2.23	40.3	2,116	9	35	JYOZ00000000
*S. sanguinis* VT517	20	2.15	41.6	2,008	4	53	JYPA00000000

a Assembly was post-assembled with a reference genome by MyPro.

The oral streptococcal strains vary in genome size, number of coding sequences (CDSs), and number of rRNAs and tRNAs (Table 1). Each nearly 2-Mb streptococcal genome contains an average G+C content of 40.8%, 1,916 CDSs, 10.9 rRNAs, and 57.9 tRNAs.

### Nucleotide sequence accession numbers.

These draft genome sequences have been deposited in GenBank under the accession numbers given in Table 1. The versions described in this paper are the first versions.
